# Rationale and design of the Baylor Infant Twin Study—A study assessing obesity‐related risk factors from infancy

**DOI:** 10.1002/osp4.463

**Published:** 2020-11-11

**Authors:** Shabnam R. Momin, Mackenzie K. Senn, Scott Buckley, Neil R.M. Buist, Manisha Gandhi, Amy B. Hair, Sheryl O. Hughes, Kelly R. Hodges, William C. Lange, Maria A. Papaioannou, Mimi Phan, Robert A. Waterland, Alexis C. Wood

**Affiliations:** ^1^ Department of Pediatrics USDA/ARS Children's Nutrition Research Center Baylor College of Medicine Houston Texas USA; ^2^ Research Prototypes Portland Oregon USA; ^3^ Department of Pediatrics Oregon Health & Science University Portland Oregon USA; ^4^ Department of Medical Genetics Oregon Health & Science University Portland Oregon USA; ^5^ Department of Obstetrics and Gynecology Division of Maternal‐Fetal Medicine Texas Children's Hospital Houston Texas USA; ^6^ Department of Pediatrics Section of Neonatology Baylor College of Medicine Texas Children's Hospital Houston Texas USA; ^7^ Department of Obstetrics and Gynecology Division of Gynecologic and Obstetric Specialists Baylor College of Medicine Houston Texas USA; ^8^ Department of Mathematics Indiana University Southeast New Albany Indiana USA

**Keywords:** design paper, epigenetics, infants, obesity, temperament, twin study

## Abstract

**Background:**

Early childhood (0–3 years) is a critical period for obesity prevention, when tendencies in eating behaviors and physical activity are established. Yet, little is understood about how the environment shapes children's genetic predisposition for these behaviors during this time. The Baylor Infant Twin Study (BITS) is a two phase study, initiated to study obesity risk factors from infancy. Data collection has been completed for Phase 1 in which three sub‐studies pilot central measures for Phase 2. A novel infant temperament assessment, based on observations made by trained researchers was piloted in **Behavior Observation Pilot Protocol** (BOPP) study, a new device for measuring infant feeding parameters (the “orometer”) in the **Baylor Infant Orometer** (BIO), and methods for analyzing DNA methylation in twins of unknown chorionicity in **EpiTwin.**

**Methods:**

EpiTwin was a cross‐sectional study of neonatal twins, while up to three study visits occurred for the other studies, at 4‐ (BOPP, BIO), 6‐ (BOPP), and 12‐ (BOPP, BIO) of age. Measurements for BOPP and BIO included temperament observations, feeding observations, and body composition assessments while EpiTwin focused on collecting samples of hair, urine, nails, and blood for quantifying methylation levels at 10 metastable epialleles. Additional data collected include demographic information, zygosity, chorionicity, and questionnaire‐based measures of infant behaviors.

**Results:**

Recruitment for all three studies was completed in early 2020. EpiTwin recruited 80 twin pairs (50% monochorionic), 31 twin pairs completed the BOPP protocol, and 68 singleton infants participated in BIO.

**Conclusions:**

The psychometric properties of the data from all three studies are being analyzed currently. The resulting findings will inform the development of the full BITS protocol, with the goal of completing assessments at 4‐, 6‐, 12‐, and 14‐month of age for 400 twin pairs.

## 1 INTRODUCTION

The global prevalence of pediatric obesity remains high, with infancy widely recognized as a critical time for obesity prevention.^1^ Over one‐fourth of the US children have overweight (sex‐ and age‐specific BMI percentile ≥85th) or obesity (≥95th percentile) by 2 years of age.^2^ Tendencies for eating behaviors and activity levels, are established during this time,^1^, ^3^ and robust associations exist between rapid weight gain in infancy and subsequent obesity.^4^, ^5^, ^6^ Yet, recently the National Institutes of Health recognized that the majority of prevention programs are designed for school aged children and raised concerns that little is understood about the factors that contribute to obesity risk in infancy.^7^

The Baylor Infant Twin Study (BITS) will address this concern, conducting cognitive, behavioral, and biological assessments on children from four months of age. Initially, BITS will focus on associations between infant temperament from four months of age with eating behaviors in both infancy and toddlerhood, with the goal of examining gene‐environment interplay underlying any associations. Phase 1 has now been completed and consists of three separate studies: The Behavior Observation Pilot Protocol (BOPP), EpiTwin, and the Baylor Infant Orometer (BIO) study.

### 1.1 The Behavior Observation Pilot Protocol

Infant temperament is emerging as a robust correlate of weight status from infancy.^8^ However, the specific temperament constructs which contribute to obesity risk are poorly understood. Studies examining this issue have mostly measured infant temperament via parent ratings.^9^ Parent‐ratings of child temperament have well‐described forms of measurement error, including “contrast effects” which are specific to ratings of temperament in infant twins^10^ and the tendency for maternal perceptions of the child's weight status to influence her assessment.^11^ A critical advancement of BITS will be the inclusion of a measure of child temperament based on objective observations made by trained researchers, which will be developed using data collected as part of BOPP.

At the initiation of BITS in 2016, we were not aware of any observation‐based measures which assessed several temperament constructs simultaneously and were validated for use in infants as young as 4 months of age. The goal of BOPP was to assess the psychometric properties of two protocols which could assess several temperament domains within a single observation period, when conducted on children as young as 4 months of age. One protocol was an adapted version of the infant (pre‐locomotor) version of the Laboratory Temperament Assessment Battery (Lab‐TAB‐P^12^), while the other utilized a stimulus‐response based assessment.^13^

Lab‐TAB‐P is designed for children from 6 to 12 months of age designed to elicit responses related to six temperament constructs: fearfulness, anger, sadness, positive affect, interest/persistence, and activity level. The Lab‐TAB‐P protocol is appealing for use in Phase 2 of BITS as there are analogous versions of the protocol available for children up to 5 years of age.^14^ However, several modifications were made to the Lab‐TAB‐P protocol to accommodate the relative lack of motor control in infants at the age of the first visit (4 months) compared to those 6 months and older.

The stimulus‐response assessment counted 31 behaviors, which when aggregated assess six temperament domains: behavioral approach, avoidance, orientation toward the mother, disengagement of attention, self‐stimulation, and self‐soothing. While this assessment offers the advantage of having been developed for children as young as 3 months of age, there is only one published use of the protocol, of which we are aware, and there is no version suitable for children over 12 months of age.^13^ The final temperament observation used in Phase 2 of BITS will be based on the measures of internal consistency, inter‐rater reliability, and test‐rest reliability across two situations (the lab and the home) and time (4 and 6 months of age) achieved in BOPP for each protocol.

### 1.2 Baylor Infant Orometer

Recent behavioral research on the development of obesity in very young children recently has focused more on *how* a child eats, rather than *what* they eat. The “how” of child eating encompasses several dimensions of eating which collectively describe *what*, *when,* and *how much* children eat (“eating behaviors”). Eating behaviors are heritable phenotypes and show robust associations with childhood weight status (see Wood et al.^15^ for a recent review).

The developmental antecedents of eating behaviors may be measurable via infant nutritive sucking, which have also been linked to the development of obesity.^16^, ^17^, ^18^ Parameters related to infant nutritive sucking can be measured reproducibly via feeding devices in which nipple flow resistance is controlled.^19^ Early devices typically measured five sucking‐related parameters which are highly correlated: total intake, total number of sucks, overall sucking rate, sucking rate within bursts, and mean suck pressure.^16^ More recently, an advanced device (the “Orometer”^20^) with an accompanying data analysis system (“Suck Editor”^20^) has been developed which measures individual differences in infant sucking via 36 related parameters representing eight broader domains: suck number, vigor, endurance, irregularity, frequency, variability, and bursting, as well as inter‐suck “rest” parameters.^20^, ^21^ BIO data extended the existing orometer data available on pre‐ and full‐term neonates^20^, ^21^ and collected the first longitudinal orometer data on infants at ages four and twelve months in order to establish which aggregated variables will be used in BITS Phase 2, based upon factor analyses, and psychometric measures including temporal stability.

### 1.3 EpiTwin

The developmental origins hypothesis posits that risk of obesity and other chronic diseases is partially set during critical periods of prenatal and early postnatal development.^22^ Individual differences in DNA methylation are a lead candidate mechanism to explain associations between the fetal environment and the entrainment of adiposity.^23^ DNA methylation is typically assessed via saliva in twin studies. However, about 75% of MZ twins share a single placenta (monochorionicity) and therefore have intermingled circulation during fetal development. The goal of EpiTwin is to examine whether there is “cross pollination” of hematopoietic stem cells in utero which results in the DNA of two monochorionic MZ twins being more epigenetically similar in a tissue‐dependent manner,^24^ causing DNA methylation in peripheral blood of two monochorionic MZ twins to be more similar than that in other tissues. Associations between chorionicity and “epigenetic supersimilarity” will also be evaluated.^25^ Accordingly, EpiTwin data will help the BITS team identify which tissues may be most suitable for studying DNA methylation in infant twins of unknown chorionicity for Phase 2.

## 2 MATERIALS AND METHODS

The targeted sample and measures collected for each of BOPP, BIO, and EpiTwin, and those planned for BITS, are listed in Table 1.

**TABLE 1:** Summary of study designs

	EpiTwin	BOPP	BIO	BITS (planned)
Target sample	Monozygotic twins	Twins ages 4 months	Singletons ages 4 months	Twins ages 4 months
Sample size (baseline)	40 twin pairs (20 monochorionic; 20 dichorionic)	31 twin pairs + 4 singletons	68 singletons	400 twin pairs
Study location(s)	Hospital (TCH)	Laboratory	Laboratory	Laboratory
		Home		
Ages	0–4 months	4,6,12 months (corrected)	4, 12 months (corrected)	4,6,12, 24 months (corrected)
Biological samples	Hair follicles	‐	‐	Urine
	Finger nails			Stool
	Urine			Sample for DNA methylation [tbd]
Assays	Blood	‐	‐	DNA methylation
	Cheek swabs			DNA sequencing
	DNA methylation			Microbiome
	DNA sequencing			Metabolome
Body composition	‐	Height	Height	Height
		Weight	Weight	Weight
		DXA	DXA	DXA
				PEA‐POD
Behavior observations	‐	Modified Lab‐TAB (temperament)	Modified Lab‐TAB (temperament)	Orometer (feeding)
		Stimulus‐response (temperament)	Novel food observation^a^ (feeding)	Actigraphy (activity level)
		Actigraphy (activity level)		Novel food observation^a^ (feeding)
		Novel food observation^a^ (feeding)		Observation [tbd] (temperament)
Questionnaires	‐	Demographics	Demographics	Demographics
		BEBQ, CEBQ‐T (eating)	BEBQ, CEBQ‐T (eating)	BEBQ, CEBQ‐T (eating)
		IBQ/CBQ (temperament)	IBQ/CBQ (temperament)	IBQ/CBQ (temperament)
Other information	Chorionicity	Chorionicity	‐	Zygosity
	Zygosity			

Abbreviations: BEBQ, Baby Eating Behavior Questionnaire^26^; BITS, Baylor Infant Twins Study; BOPP, Behavior Observation Pilot Protocol; CBQ, Child Behavior Questionnaire^27^; CEBQ‐T, Child Eating Behavior Questionnaire for Toddlers^28^; CNRC, Children's Nutrition Research Center; DXA, Dual‐Energy X‐Ray Absorptiometry; IBQ, Infant Behavior Questionnaire^27^; tbd, to be decided; TCH, Texas Children's Hospital.

^a^ 12 months of age only.

### 2.1 Sample and recruitment

#### 2.1.1 Behavior Observation Pilot Protocol

Twins were recruited for BOPP via four routes: (1) from women in prenatal or postnatal clinics at Texas Children's Hospital (TCH), via flyers distributed from clinicians; (2) approaching women in their postpartum room at TCH after identifying twin births via TCH electronic medical records (EMRs); (3) advertisements posted in the websites and Facebook pages of local groups such as “Bellaire Moms of Multiples”; and (4) fliers posted in the waiting room at Texas Children's Pediatrics clinics; and (5) invitation to join BOPP after participation in EpiTwin. Interested families were invited to contact the research team for more information and to schedule a screening phone call. Exclusion criteria included infants from a higher order multiple, with a birth weight less than 1800 grams, with major congenital anomalies and/or with parents with inadequate English to understand the study protocol and give informed consent.

#### 2.1.2 Baylor Infant Orometer

Children were recruited in the same manner as for BOPP, with the exclusion of routes (2) and (5) that is not via post‐partum contact at TCH or via participation in EpiTwin. Inclusion criteria included the “regular” use of a bottle (defined as at least one normal‐sized feed per day). Exclusion criteria included birth weight of at least 2267 g and parents able understand the study protocol and give informed consent between 2017 and 2019.

#### 2.1.3 EpiTwin

Mothers of twins born at TCH were identified via EMRs and approached in their postpartum hospital room for study participation, in consultation with floor nurses and obstetrician/gynecologist staff. Exclusion criteria included infants with major congenital anomalies.

*Ethical Standards (BITS/BOPP/BIO/EpiTwin*): Ethics approval was received for all studies from the Institutional Review Board at Baylor College of Medicine (BITS/BOPP: H‐36097; BIO: H‐40416; EpiTwin: 37,359). The authors attest that all procedures contributing to this work comply with the ethical standards of the Helsinki Declaration of 1975, as revised in 2008.

### 2.2 Procedures

#### 2.2.1 Behavior Observation Pilot Protocol

When the twins were 4 months of age (corrected for gestational age), infants and their main caregiver(s) visited the Metabolic Research Unit (MRU) at the Children's Nutrition Research Center (CNRC). After obtaining written assent from the parents, infants were outfitted with actigraphs and participated in separate temperament observations and assessments of body composition. During the temperament observation, the caregiver completed questionnaires on demographics and infant behaviors. Infants were allowed a break, as needed, between the assessments. The protocol lasted approximately 4 h. Within 1 week, research assistants visited the twins in their home where they repeated the temperament observation. This procedure was repeated at 6 months of age. At 12 months of age, twins and their caregivers were invited to the research center to participate in a final temperament observation and a feeding observation. No home visit occurred at 12 months of age.

#### 2.2.2 Baylor Infant Orometer

When the children were 4 months of age (corrected for gestational age), infants and their main caregiver(s) visited the MRU at the CNRC. After obtaining written assent from the parents, infants completed a feeding observation (via orometer), a temperament observation, and an assessment of body composition. The caregiver completed questionnaires on demographics and infant behaviors. The specific order of the tasks was varied dependent on the needs of the infant—if the infant was excessively fussy, the caregiver(s) were offered a quiet room in which to let the infant sleep in the presence of the caregivers while they completed the questionnaires. The protocol lasted approximately 2.5 h. At 12 months of age, infants and their caregivers were invited to the CNRC to repeat the protocol, with the addition of a novel food assessment.

#### 2.2.3 EpiTwin

After receiving written assent from at least one parent, the infants had biological samples collected by a trained research assistant while the twins were in the neonatal intensive care unit (NICU) or in the postpartum recovery room. Both members of each twin pair had their samples collected on the same day, although different tissues were sometimes collected on different days.

### 2.3 Biological measures

#### 2.3.1 Buccal swab (EpiTwin)

DNA Genotek OC‐175 sample kit was used to gently swab between infant's cheek and gums forward and back 10 times on each side of the mouth. The swab was then placed back into tube, screwed tightly, and shaken up and down 10 times. The tube contained a solution which allows the DNA to stay at room temperature until DNA isolation using standard method.^29^

#### 2.3.2 Hair follicles (EpiTwin)

From an area on the head, precleaned using alcohol swabs, sterile tweezers were used to pluck hairs, including the follicle bulb at the base of hair strand and root. The hairs were placed in a sterile, 1.5 ml clear tube and placed on ice (max ∼ 1 h) then transferred to −80°C until DNA isolation.

#### 2.3.3 Urine (EpiTwin)

Urine samples were collected using the pediatric urine collector bag, with adhesive closure, placed over the infant's genitals. The bag remained in place until sample collection was complete. Urine was immediately combined with a urine conditioning buffer, after which it is stable for up to 6 days until DNA isolation (Extract‐ALL Urine DNA Kit, Zymo Research).

#### 2.3.4 Peripheral blood cells (EpiTwin)

A small heel prick was administered by phlebotomist. PE 226 Spot Saver Card (PerkinElmer Inc) was used to collect a single sample of blood. Each well holds 75–80 µl of sample. The QiaAmp DNA Blood Mini Kit (Qiagen) was used to isolate DNA from blood.

#### 2.3.5 Nail clippings (EpiTwin)

Parents clipped infants' fingernails (preferably) or toenails (if necessary) using new, alcohol‐sterilized standard safety first nail clippers. The nail clippers were fitted with tape enclosures to retain the clippings. The nail clippings were transferred to a sterile 1.5 ml tube and stored at −80°C, then washed several times in EDTA before DNA isolation.

### 2.4 Assay‐based measures

#### 2.4.1 Zygosity (EpiTwin)

DNA sequencing has yet to be completed, but zygosity will be determined via genotyping microarray using either H3Africa chip or Infinium global screening array.

#### 2.4.2 DNA methylation (EpiTwin)

Genomic DNA was bisulfite‐modified (EZ DNA Methylation‐Direct kit, Zymo Research), amplified by one‐round of PCR using locus‐specific primers, and sequenced on a Qiagen MD Pyrosequencer, by our standard protocol.^30^, ^31^ Prior to use, the accuracy and sensitivity of all of our pyrosequencing assays using human genomic methylation standards was validated by our team (Harris et al., 2010).^32^

### 2.5 Temperament observations

Temperament observations were conducted in the MRU at the CNRC (the “lab”—BOPP/BIO) and at the infants' homes (BOPP only). The MRU comprises an observation room and a control room. The behavior observations took place in the observation room, which was well equipped with 4 cameras to capture views from all angles. Furniture in the room consisted of a table, high chair(s), a car seat, a sofa and/or rocking chair (as requested by the mother), and a TV screen with camera mounted on the top of the screen. The control room is equipped with computer‐assisted controls for the observer to focus and manage the cameras and record the observations. During the behavior observations, the infant was seated in a highchair with a tray table. Video cameras are adjusted to capture the car seat “head on” such that the infants eye gaze, hand and feet and head movements can be captured. The field of vision is large enough to encompass kicking and pointing. A digital clock with seconds is visible to the experimenter, but not the child.

At home observations mostly took place in the participant's family room. The infant was seated in a highchair with a tray table. Two cameras were mounted on tripod stands and adjusted to capture the infant's gaze, hands and feet, and head movements.

#### 2.5.1 Modified Lab‐TAB (BOPP/BIO)

At 4‐, 6‐, and 12‐month of age four (BOPP) or two (BIO) modified Lab‐TAB episodes were conducted: for BOPP and BIO these were “puppet game” and “slide show”, and for BOPP only, the “peek‐a‐boo” and “jungle gym” episodes (“jungle gym” was modified from the original “block” episode) were also conducted.

#### 2.5.2 Stimulus‐response assessment (BOPP)

The infant was placed in an infant car seat with a tray table with a plain cover (such as a white blanket to avoid distractions). The infant was lightly restrained using the chest clip only, to stop “slumping” and so reduce the need for the child to be picked up unnecessarily. The caregiver was seated to the infant's right, approximately 60 cm away, out of the child's line of vision, and asked to refrain from verbally interacting with the infant. A trained research assistant started the protocol by reading from a prepared script. Three stimuli were presented to the infants for 20 s in the following sequence: a checkerboard was shown out of reach (8–10 inches from face), a bell was rung out of reach but within sight, and a rattle was shaken out of reach but within sight after which it is placed on the tray table within infant's reach for 30 s. This procedure was repeated three times.

### 2.6 Mechanically assessed activity level

#### 2.6.1 Actigraph assessments (BOPP)

Three ActiGraph GT3X + accelerometers, were placed on the infants: one on each leg of the baby (below knee and above the ankle) and another on the waist.

### 2.7 Feeding observations

#### 2.7.1 Orometer (BIO)

Ahead of time, the caregiver was asked how they “normally” feed their child, in terms of what the infant and caregiver usually sit in (e.g., chair, sofa, and bed), and how they are positioned relative to one another during a typical daytime feed. The team then attempted to replicate the set up in observation room. Caregivers were asked to supply milk, which was placed in a standardized, presterilized baby bottle (6 ounce Playtex BPA free VentAire standard bottle), fitted with a sterilized Enfamil standard flow soft disposable nipple which was discarded after each use. Finally, a chamber which housed the orometer pressure sensor was attached to the bottle and was connected to the computer via wire. The families were allowed as much time as they needed to complete the feeding.

#### 2.7.2 Novel food observation (BOPP/BIO)

At 12 months infant's acceptance/rejection to novel food was assessed in the MRU, CNRC observation room using the modified version of the protocol developed by Moding and Stifter.^33^ Both infant twins were each placed in a high chair across from their mother, with the same set up for singletons in BIO. The four cameras were focused on the infants and the mother. The experimenter gave the mother a plastic spoon and a small bowl containing one of the foods that was previously identified by the mothers as novel to her infant. The novel foods used in BOPP were quinoa, couscous, or farrow. These foods were later expanded to include fine bulgar, kiwi, mango, papaya, starfruit, and pineapple for BIO. The mother was instructed to feed both infants as she would normally feed them at home. The mother was instructed to continue feeding as long as she wished (if the infant accepted the food), or to keep feeding the infant(s) until he/she refused the food three times. To ensure that infants did not become satiated, and so their initial response to the food was captured, the task was ended after 3 min unless the infant rejected the food three times before the 3‐min mark. The experimenter observed the infant(s) from the control room. Once infants rejected the food three times, which included turning away, swatting the spoon, or refusing to take a bite of the food, the experimenter reentered the room, and asked the mother to stop feeding her infant(s).

### 2.8 Body composition

#### 2.8.1 Dual‐Energy X‐Ray Absorptiometry (BOPP/BIO)

The infant was swaddled and lays face‐up while a X‐ray detector source scans over the body. Raw scan data were converted to an image and a quantitative measurement of the bone and body tissues, resulting in estimates of total body bone, fat, and lean mass.

#### 2.8.2 Length and weight measurements (BOPP/BIO)

Recumbent length was measured using an infant stadiometre with a fixed head piece and horizontal backboard, and an adjustable foot piece; weight was assessed using a Sartorius weight scale.

### 2.9 Questionnaire‐based assessments

#### 2.9.1 Child temperament (BOPP/BIO)

Temperament was assessed via caregiver‐report, using the Infant Behavior Questionnaire (IBQ^27^)—very short version when the children were less than 12 months of age, and the Child Behavior Questionnaire (CBQ^27^)—very short version when the children were 12 months of age or older. The IBQ and CBQ measure 14 and 15 temperament dimensions, respectively, with considerable overlap in the dimensions measured. Common to both measures are the dimensions of approach, high intensity pleasure, smiling and laughing, activity level, perceptual sensitivity, fear, falling reactivity/rate of recovery from distress, ow‐intensity pleasure, and soothability. The IBQ additionally measures vocal reactivity, sadness, distress to limitations, and duration of orienting, while the CBQ additionally measures attentional focusing, anger/frustration, discomfort, impulsivity, impulse control, and shyness.

#### 2.9.2 Child eating behaviors (BOPP/BIO)

The caregiver reported child eating behaviors via the Baby Eating Behavior Questionnaire (BEBQ^26^) at 4 and 6 months of age and the Child Eating Behavior Questionnaire for Toddlers (CEBQ‐T^28^) at 12 months of age. Like with the IBQ and CBQ, there is considerable overlap in the eating behaviors measured between the BEBQ and CEBQ. Both questionnaires assess enjoyment of food, food responsiveness, slowness in eating and satiety responsiveness. The CEBQ additionally assesses four eating behaviors difficult to discern in children under 12 months of age: emotional overeating, desire to drink, emotional undereating, and food fussiness.

#### 2.9.3 Demographic information (BOPP/BIO)

The mother completed a short questionnaire which included information on both the mother and child. Age and race/ethnicity of the mother were collected, while for infants age (days), gender, gestational age, and birth weight were collected.

#### 2.9.4 Chorionicity (EpiTwin)

At the referring centers for this study, chorionicity is routinely determined by ultrasound by experienced sonographers at 11–18 weeks' gestation, as standard medical practice for multiple gestations. Chronicity is recorded in the EMR and was retrieved by the study coordinator using TCH EPIC system.

## 3 DISCUSSION

In assessing associations between infant temperament and obesity risk, BITS will incorporate methodological innovations such as a comprehensive assessment of infant temperament based on observations made by trained researchers. As such, it has been necessary to pilot several procedures before designing the final BITS protocol via the BOPP, BIO, and EpiTwin sub‐studies conducted as Phase 1 of BITS. The data collection for these three pilot studies has been completed and analysis will soon be underway.

The analyses of the three pilot studies described above will contribute to measures used in the full BITS study. BOPP will identify a measure of infant temperament with good inter‐rater reliability, and both cross‐situation (laboratory and home) and cross‐time (between 4 and 6 months of age) stability in children as young as 4 months of age. Meanwhile BIO will identify a psychometrically sound measure of feeding behaviors in infants. EpiTwin will reveal the most suitable tissue for DNA methylation analysis when using twin pairs of unknown chorionicity.

It is anticipated that the full BITS protocol will take place in the laboratory at 4, 12, and 24 months of age and involve a single temperament observation, an eating observation, an assessment of body composition, and the collection of several tissues for analysis of DNA variation, DNA methylation, the microbiome, and metabolome. The decision to include these measures and exclude other potentially informative measures (e.g., feces for analysis of the gut microbiome, or maternal prepregnancy body weight) was made according to the balance of how reliable the measure would be against the participant burden of collecting this measure. Ultimately, BITS will take an integrative approach that includes analysis at multiple levels—including genes, cognition, microbiome, and nutrition—to identify obesity risk factors very early on in life.

## CONFLICT OF INTEREST

All authors declare they have no conflicts of interest.

## AUTHOR CONTRIBUTIONS

Alexis C. Wood, Sheryl O. Hughes, and Robert A. Waterland conceived the study design(s). Shabnam R. Momin, Mackenzie K. Senn, Manisha Gandhi, Amy B. Hair, Kelly R. Hodges, Maria A. Papaioannou, Mimi Phan, and Alexis C. Wood conducted data recruitment. All authors were involved in writing the paper and had final approval of the submitted and published versions.
